# A Multi-Theoretical and Multi-Method Family Study Approach to Preschool Inhibitory Control: Links to Working Memory, Receptive Vocabulary, Behavioral Maladjustment, and Parent Mental Health in the Context of Temperament and Executive Functioning Perspectives

**DOI:** 10.3389/fpsyg.2021.703606

**Published:** 2021-08-12

**Authors:** Jeffrey R. Gagne, Kaelyn Barker, Chi-Ning Chang, Ogechi K. Nwadinobi, Oi-Man Kwok

**Affiliations:** ^1^Department of Educational Psychology, Texas A&M University, College Station, TX, United States; ^2^KU Life Span Institute, University of Kansas, Lawrence, KS, United States

**Keywords:** inhibitory control, self-regulation, executive function, multi-theoretical, multi-method

## Abstract

Inhibitory control (IC) is defined as the executive functioning (EF) and self-regulatory temperamental inhibition of impulsive or pre-potent behavior and has been consistently linked to multiple forms of childhood cognitive and socio-emotional maladjustment including academic and learning challenges, externalizing behaviors, and attention deficit hyperactivity disorder. However, the results of relevant investigations are somewhat dependent on the method of IC assessment and the theoretical approach of the researcher. The two primary theoretical perspectives on IC are the temperament and the EF approaches, and although there is considerable overlap between these perspectives, there are some distinctions with regard to assessment and emphases on cognition vs. emotion. Therefore, investigations including both temperament and EF approaches to IC are of considerable interest and will best inform future education, prevention, and intervention efforts. This investigation examined associations between child IC, working memory (WM), receptive vocabulary, externalizing behavioral problems, and primary caregiver depression and anxiety symptoms using a family study design. The sample was composed of 99 families with two typically developing preschool children (*n* = 198; 2.5–5.5 years old; *M* = 3.88, *SD* = 1.04) and one primary caregiver/parent. Child IC was assessed using a multi-method approach consisting of one parent-rated questionnaire, three independent observer rating subscales, two videotaped in-person laboratory temperament episodes, and an EF Stroop task. Child WM and receptive vocabulary were measured in the laboratory using standard assessment techniques, and the remaining measures were parent-reported. Male child participants had significantly higher levels of observer-rated hyperactivity and impulsivity, and females had higher levels of observer-rated attention and Stroop-assessed IC. Correlational results showed that excepting IC-Stroop and a snack delay task, all IC measures were significantly correlated. All IC measures except snack delay were positively correlated with WM, and with receptive vocabulary (except Lab-TAB snack delay and observer-rated hyperactivity), and WM and receptive vocabulary were also positively correlated. All IC variables, WM, and receptive vocabulary were significantly related to externalizing behavior problems. Generally, children with higher IC, WM, and receptive vocabulary had lower levels of behavioral maladjustment. Lower parent-rated IC and higher levels of externalizing behavior problems were positively associated with maternal depression and anxiety (lower receptive vocabulary level was related to depression only). Employing structural equation modeling (SEM) analyses, we further examined the interrelationships among IC temperament variables, IC-Stroop, WM, and receptive vocabulary, controlling for age, gender, externalizing behaviors, maternal depression and anxiety, and the parent-rater variance (the multi-method effect). The results of our hypothesized model showed that the IC Temperament factor, composed of the six temperament IC measures, showed a positive effect on receptive vocabulary, while the IC-Stroop positively predicted WM. The IC Temperament factor and IC-Stroop were positively correlated with each other, and the IC Temperament factor, IC-Stroop, WM, and receptive vocabulary were positively related to age. The IC Temperament factor was also associated with fewer externalizing behavior problems, maternal depression had a negative effect on receptive vocabulary, and females showed lower levels of WM and receptive vocabulary than males. Overall, the IC Temperament factor and other covariates together accounted for 22.5% of the variance in vocabulary, whereas IC-Stroop and other controlled variables could explain 49.8% of the variance in WM. These findings indicate that theoretical perspectives (in this case temperament and EF IC contexts) and the different types of assessments used are crucial considerations when interpreting the results of studies of early childhood IC. Although most assessments of IC were associated with the outcomes under study, we found specific associations between temperament measures of IC and receptive vocabulary as well as externalizing, and IC-Stroop and WM. In addition, maternal depression had an effect on receptive vocabulary, emphasizing the developmental importance of family environment in preschool. These findings are relevant to the field of child development because they address several important questions about child EF and self-regulation. 1. Do temperament and EF conceptions of IC differentially predict outcomes? 2. How does the way we measure IC from the EF and self-regulation/temperament perspectives impact our conceptualizations of these important constructs? 3. How can we reconcile the various ways different disciplines define IC and their independence/overlap? 4. How can multi-method and multi-disciplinary perspectives and data collection approaches be combined to better understand both the temperament and EF conceptions of IC? Future studies with this sample will employ this multi-theoretical and multi-method approach on assessment in preschool to predict temperament, EF, and behavioral and academic adjustment in elementary school longitudinally.

## Introduction

The two primary theoretical perspectives on inhibitory control (IC) are the temperament and executive functioning approaches. While these perspectives are similar, there are some distinctions of note. Temperament is described as – emerging individual differences in behavioral and emotional development that emerge in infancy, are moderately stable throughout development, and have a biological or genetic basis (Rothbart and Bates, 2006; Zhou et al., 2012). Many psychologists emphasize child temperament because of its strong associations with adult personality as well as maladaptive traits, such as psychopathology in childhood (Goldsmith et al., 2004; Caspi et al., 2005). While temperament researchers focus primarily on socio-emotional development, Rothbart included aspects of cognitive development, specifically executive attention in her temperament theory (Rothbart and Bates, 2006). Rothbart’s theoretical perspective maintains that IC as a distinct temperament dimension that is subsumed by the larger domain of effortful control (Rothbart et al., 1994; Rothbart and Bates, 2006). Effortful control (EC) is “the efficiency of executive attention, including the ability to inhibit a dominant response, to activate a subdominant response, to plan, and to detect errors” (Rothbart and Bates, 2006, p. 129). Therefore, according to the temperament perspective, IC is the inhibition of impulsive or pre-potent behavior, typically under the conditions of expectation or instruction (Rothbart, 1989; Kochanska et al., 1996).

IC involves the control of impulses, and basic examples include avoiding eating a snack for an extended period, similar to Mischel’s renowned “marshmallow task” (Metcalfe and Mischel, 1999), and being successful on similar delay tasks often used in temperament investigations of IC. Cognitive psychologists and neuroscientists also describe the phenomenon of IC, but as a component of executive functioning (EF) rather than as a dimension of temperament. EFs are mental processes related to cogitating, concentration, and planning, and EF scholars refer to IC as the capacity to regulate thinking, behavior, and in many cases emotion to sustain attention and resist impulsive thoughts and behaviors (Diamond, 2013). Generally, IC in children is assessed from the EF perspective using Stroop and other similar tasks in order to determine the child’s ability to engage executive control. Therefore, school age children are typically the focus for the EF approach on IC. The temperament approach typically emphasizes earlier assessment of IC with affective components that tend to be less cognitively challenging than standard EF assessments of IC that rely more on attentional focusing.

The early development of IC is a critical area of study in developmental science. Researchers have found that children with low IC have a higher risk of behavioral maladjustment, such as externalizing behavior and attention deficit hyperactivity disorder (ADHD; Eisenberg et al., 2001, 2004; Goos et al., 2009; Gagne et al., 2011). This pattern f findings is consistent across both executive functioning and temperament researchers studying IC and behavioral maladjustment in childhood (Nigg et al., 1999; Olson et al., 1999; Eisenberg et al., 2001, 2005; Murray and Kochanska, 2002; Riggs et al., 2003; Hughes and Ensor, 2008; Gagne et al., 2011). Children with early behavior problems have increased risk for several poor developmental outcomes related to health and education (Saudino et al., 2008; Gagne, 2017).

A related aspect of executive functioning that developmental researchers study in childhood is working memory (WM). WM is described as the capacity to cognitively retain information that is no longer being directly perceived and manipulate it in some way (Baddeley and Hitch, 1994; Smith and Jonides, 1999; Diamond, 2013). Therefore, WM is key to the interpretation of events that occur in real time because one must recall a recent occurrence and relate it to a current one. This is a fundamental component to comprehending written and oral language and for mentally computing math (Diamond, 2013). WM is also very important for IC functioning, as each often supports the functioning of the other (Diamond, 2013). For instance, one must retain the rules or instructions (WM) to inhibit impulses counterintuitive to what is necessary to accomplish a goal (IC). Alternately, one must focus attention through the inhibition of distractions to best interpret the rules for a specific goal or task. Therefore, both executive functions provide support for the other (Diamond, 2013). Studies have shown strong links between EF conceptions of IC and WM in young children, and several have indicated that confirmatory factor analyses of the two constructs yield a single common factor (Wiebe et al., 2008, 2011). However, other analyses have yielded a two-factor solution (Lerner and Lonigan, 2014; Usai et al., 2014; Panesi and Morra, 2020) with some scholars suggesting the associations between IC and WM are strongest in younger children, and as children age, the two constructs become more distinct from one another (Lerner and Lonigan, 2014).

Vocabulary is a basic building block for many school-related subjects, as it is significantly associated with listening and reading comprehension skills in childhood (Cain et al., 2004; Cromley and Azevedo, 2007; Lynch et al., 2008; Florit et al., 2009; Language and Reading Research Consortium et al., 2019). Many also propose that vocabulary may be related to the development of EFs, as language improves children’s ability to think, learn, use goal-oriented rules, and trigger deliberate discipline of their actions according to the iterative reprocessing model and cognitive complexity and control theory (Zelazo et al., 2003; Zelazo, 2015; Schmitt et al., 2019). Although it is generally accepted that vocabulary skills support cognitive development including EFs, interpretations of this research are somewhat mixed in findings due to varying levels of complexity in vocabulary skills assessed (Schmitt et al., 2019).

Since IC and WM are the primary EFs of childhood, the early development of these skills and the acquisition of vocabulary overlaps. For example, research indicates that WM shows individual differences in childhood that strongly predict performance in attention, reasoning, and comprehension that may be relevant for IC (Engle and Kane, 2004; Unsworth and Engle, 2007; Unsworth and Robison, 2017). In addition, individuals with low WM capacity along with low attentional control report increased distractedness, absent-mindedness, and mind wandering (Unsworth et al., 2012). McClelland et al. (2007) showed that more behaviorally regulated children, with higher levels of IC (Blair, 2002), obtained significantly higher levels in vocabulary with these results pointing toward a crucial relation between behavioral regulation and school readiness (Bronson, 2000; Blair, 2002). This research also found that increases in self-regulation across the academic year revealed greater advances in vocabulary as well as emergent literacy (McClelland et al., 2007). Similarly, Wolfe and Bell (2007) found that high composite scores of WM and inhibitory control were associated with the highest levels of language achievement.

A multi-method approach to IC measurement can offer a clearer representation of early emerging IC and associations with school readiness and behavioral maladjustment. Although EF tasks tapping IC are used in research on preschoolers, these measures can be relatively difficult for younger children. More basic behavioral measurement of IC in preschool is also possible by employing inhibition and delay tasks during a videotaped assessment of temperament in the laboratory. The preschool version of the Laboratory Temperament Assessment Battery (Lab-TAB^1^) contains multiple IC episodes that have been used in earlier studies (Gagne and Saudino, 2010, 2016; Gagne and Goldsmith, 2011; Gagne et al., 2011). Though both EF and temperament tasks are available for IC assessment in preschool, many investigations of early emerging IC rely on parent-rated questionnaires as the principal assessment modality. Parent-rated questionnaires are reliable and valid but are prone to rater bias, including contrast effects in studies of family members (Saudino, 2003). In some cases, parent ratings of IC may show stronger rater covariance with parent-rated behavior problems (Gagne et al., 2018). Therefore, incorporating parent ratings as well as EF and temperament tasks will provide a more comprehensive assessment approach and will allow investigators to account for shared method variance, better identifying the primary IC variables links to outcome variables (Podsakoff et al., 2003).

Although in some cases, different measures of IC confer different patterns of results in predicting variables of interest, there is some evidence that EC and EF measures of child IC tap a common factor of self-regulation (Lin et al., 2019; Tiego et al., 2019; Kälin and Roebers, 2021). In addition, some researchers have viewed EFs in general as either a unitary or multi-dimensional construct. For example, Garon et al. (2008) describe the component EFs as being subsumed under an executive attention system, with elementary forms of the core EF s present in preschool. Others have separated more complex EF and IC tasks into those that require effortful or automatic inhibition, overlapping with EF and temperament measurement conceptions of IC, respectively (Johnson et al., 2003). A recent selective review of EF terminology and methodology argues that further conceptual clarity is required regarding EFs and IC and inhibition and suggests that task appropriateness may depend on study goals and the ages of the participants (Morra et al., 2018). We agree with this viewpoint and further argue for the inclusion of temperament measures in IC assessment during preschool. Employing a multi-method and multi-informant approach allows researchers to study if IC measures are best analyzed separately or by using a common factor approach.

In addition to employing multiple measures of IC, it is also important to consider the family context. Family studies that include parent and sibling data permit researchers to examine the influence family members have on developmentally significant traits in childhood. Many studies of individual differences in child IC emphasize child behavior. However, the family environment is also germane to the early development of IC, and parent traits, such as neuroticism, emotional or affective style, parenting style, substance use, and depression and anxiety symptoms, are often investigated. A family study including both parent and child variables (such as a multi-method perspective on IC assessment) can clarify the development of preschool IC and links to WM and vocabulary. Results from a comprehensive study such as this can inform child and family assessment, allowing for better recognition of risk factors, and improve interventions.

The current study examined relations between multiple measures of IC, as well as unitary measures of WM and receptive vocabulary in preschool-aged siblings. Additionally, we investigated how these three preschool abilities were related to early emerging child behavior problems and maternal mental health symptoms. Instead of assessing one child exclusively, two preschool-aged siblings from a family were recruited, increasing the power to detect effects as opposed to studying singletons (Krull, 2007). The inclusion of siblings in analyses accounts for IC, WM, and receptive vocabulary variance caused by dyadic differences, and differences in variance due to family means, sources of variance that are confounded in studies of single children (Spann and Gagne, 2016). The proposed SEM analyses account for these between and within sibling and family effects. We predicted lower levels of child IC, WM, and receptive vocabulary would be significantly correlated, associated with externalizing behavior problems, and we were interested in seeing if these results would be consistent across multiple assessments of child IC. Based on our previous findings (Gagne and Saudino, 2010, 2016; Gagne and Goldsmith, 2011; Gagne et al., 2013a, 2018), girls and older children were hypothesized to have higher IC and be at less risk for externalizing problems. Although performance gender differences are not evident for WM, there is some evidence of neurofunctional gender differences (Hill et al., 2014). Therefore, we tested for gender differences in WM as well. The analyses accounted for child gender and age, and the depressive and anxiety symptoms of the primary caregivers (virtually all mothers). Based on prior findings (Gagne and Saudino, 2010, 2016; Gagne and Goldsmith, 2011; Gagne et al., 2013a), boys and younger children were predicted to have lower IC and be at higher risk for externalizing behavior problems. In addition, caregiver depressive symptoms and trait anxiety were both associated with externalizing behavior problems in recent results in our sample (Gagne et al., 2018). Several other studies have shown similar findings regarding parental psychopathology and child temperament and EF (Ventura and Stevenson, 1986; Edhborg et al., 2000; Biederman et al., 2001; Pesonen et al., 2006; Gagne et al., 2013b). Therefore, we predicted that maternal depressive and anxiety symptoms would be related to child externalizing behavior problems.

## Materials and Methods

### Participants

The participants in the TEXAS Family Study, which investigated child temperament, behavior problems, executive function, and relevant parent and family variables, served as the sample for the current research. Ninety-nine families with two typically developing preschool children (*n* = 198) and one parent participated in the study. The age range for these children was 2.5 to 5.5 years old (*M* = 3.88, *SD* = 1.04). There were 102 males (mean age = 3.79, *SD* = 0.99) and 96 females (mean age 3.97, *SD* = 1.08), 57 full sibling pairs, 10 identical twin pairs, and 32 fraternal twin pairs included in the sample. The racial distributions included a predominantly white sample (84% of children; 88% of mothers; and 87% of fathers). Other races included in the sample were Hispanic or Latino (13% of children; 7% of mothers; and 8% of fathers), multiracial (11% children; 5% mothers; and 4% fathers), and African American (4% children; 4% mothers; and 7% fathers). Less than 3% were reported as Asian American, Pacific Islander, and other races. The average household income was approximately $70,000 (Range= $20,000–$200,000) and average years of education was 15.82 years for mothers and 15.2 years for fathers (range from 8 to 22 years), respectively.

### Procedure

Recruitment information on the TEXAS Family Study is documented in Gagne et al. (2018). Online surveys were completed through SurveyMonkey (predominantly by mothers) after recruitment and screening based on child age and developmental status. There were 126 families that completed this online portion; and of these families, 100 families participated in a laboratory visit (children with any developmental disorders, such as autism, were excluded from analyses). The laboratory visits utilized different behavioral and cognitive assessments described as “fun games” for the children. Parents also filled out additional questionnaires while at the visits. There were no significant differences in demographic variables for families (parental age, years of education, and family income) that completed the laboratory option and those that just completed the online surveys, with one exception, child age. Average child age was lower for the participants with parents who completed the online phase of the study only. This occurred because some children were not within the age range of the study, and therefore were ineligible for laboratory visits. After completing the online portion of the study, participants were remunerated a $25 gift card. The laboratory visit participants were compensated an additional $50 gift card after completion of the visit. The University Institutional Review Board reviewed and approved all research procedures for this study.

### Measures

#### Inhibitory Control

A multi-method approach was used to comprehensively measure IC for this study. The measures used for this approach were a parent-rated questionnaire, multiple laboratory-based tasks, and a global observer rating completed by child experimenters.

#### Parent Ratings of Inhibitory Control

The parent report measure used was the Toddler Behavior Assessment Questionnaire-Revised (TBAQ-R; Goldsmith, 1996). The IC subscale of the questionnaire assesses child IC with a higher score indicating higher levels of IC. The TBAQ-R contains 120 items, and 13 items comprise the IC subscale. These items estimate child temperament traits by assessing the frequency of child behaviors that occurred in specific scenarios in the past month as rated by parents. TBAQ-R items are composed of a 7-point Likert scale with 1 being “never” and 7 being “always” (as well as an “N/A” option). The TBAQ-R IC subscale used in our sample had an internal consistency reliability of 0.93, consistent with Goldsmith (1996).

#### Stroop Task

A modified Stroop task was used in the laboratory in order to measure child IC based on the EF approach. The task required that the child inhibits their natural impulse response to answer task questions appropriately. The tasks were adapted for different age groups. These adaptations of the Stroop task all included an introductory phase for the control condition, which did not have any IC requirement on the child followed by the test condition. This test condition did require IC to inhibit a pre-potent response following the directions for the task (Hughes and Ensor, 2007). The first age range (children 2.5–3.5 years old) performed the baby Stroop task (Hughes and Ensor, 2005). This task required offering the child a smaller “baby” cup and a larger “mommy” cup. Researchers then asked the participant to point to either cup. This introductory phase for the task allowed the verification of the child’s ability to distinguish the two different cups. Next, the participant was told that they were playing an “opposite game.” When shown the “mommy cup,” the child would need to say “baby cup” and vice versa. The Stroop task displayed the two different stimuli cups in a pseudorandom order, where the researcher would bring one cup forward at a time. The next age range (3.5–4.5 years old) participated in the hand game (Hughes, 1998). Researchers displayed a fist and then displayed a pointing finger. The child mirrored this display for the introduction to the task followed by the test condition. The child was asked to point when the experimenter made a fist and shows a fist when the researcher pointed. A Cronbach’s alpha of 0.88 was reported for this activity (Chasiotis et al., 2006). The final age range (4.5–5.5 years of age) played the day-night task. This task asked that the child respond by saying “day” if a card with a moon and stars was presented (the “night” card). When presented with a card depicting the sun (the “day” card), the child was to respond with “night.” This was repeated for 12 trials, and the number correct was counted. High scores demonstrated higher levels of IC. The range of 0.79 to 0.93 was reported for the internal reliability of this modified Stroop assessment (Chasiotis et al., 2006; von Stauffenberg and Campbell, 2007; Rhoades et al., 2009). An 0.84 test–retest reliability score was reported for this measure (Thorell and Wåhlstedt, 2006).

#### Laboratory Temperament Assessment Battery

An in-lab standardized assessment battery was also used to measure child IC using preschool Lab-TAB episodes (see footnote 1). The Lab-TAB IC “snack delay” and “gift” episodes were used to measure IC. Coders for this behavioral data went through training to be within 90% agreement with the master coders. After that proficiency was obtained, they went on to code the behavioral data independently. To further assess coder reliability, 20 % of the sample episodes were double-coded by another coder and agreement reached over 85%. The “snack delay” task consisted of providing the child a snack typically M&M’s or goldfish and providing directions to wait for a bell to be rung before eating the snack. One snack was placed under a clear cup on a plate and the experimenter would ring a bell when it was time for the child to eat the snack. This was repeated for six trials with wait intervals of 20, 30, 0, 40, 10, and 60 s in this order. In the “gift” task, a research assistant presented a small gift-wrapped toy to the child and asked them to wait for a period of time before signaling that they were allowed to open this gift. The child was then ignored by the experimenter (the child was led to believe that the researcher is working on paper work during the task) for 2 min before given permission to open it by the instructor.

#### Post-visit Observer Temperament Rating

Post-visit ratings of child temperament were also completed by study experimenters after each laboratory visit. The scale for these post-visit ratings was based on a 23-item questionnaire estimating different aspects of temperament on a 5-point scale where 1 served as the lack of a characteristic (e.g., frustration, energy, or impulsivity) and 5 served as a high magnitude, regularity, and/or severity of such a characteristic. Previous studies of temperament conducted comparable ratings (e.g., Gagne et al., 2011) to serve as convergent validity for Lab-TAB and questionnaire measures. Attention to tasks, hyperactivity, and impulsivity (selected because of their overlap with IC) was used to assess items post-visit rating child IC scores for this study (internal consistency = 0.82).

##### Working Memory

A search task called Spin-the-Pots (Hughes and Ensor, 2005) was used to assess WM in the child participants. The game utilized several visually specific boxes that were placed on a Lazy Susan. The total boxes used for the task were determined by child’s age with 2.5–3.5-year olds using eight boxes to choose from, children ages 3.5–4.5 years old having 10 boxes, and children between the ages of 4.5–5.5 using 12 boxes. The child helped to set up the task by assisting in inserting the stickers inside the boxes. The researcher informed the participant they did not have enough stickers to place in each box. Thus, two boxes remained empty. Once stickers were placed and the boxes closed, the Lazy Susan was then covered with a cloth. Next, the Lazy Susan was spun around once and uncovered. The child was then asked to choose a box with a sticker inside. The child decided, then the Lazy Susan was covered again. The procedure continued until the stickers were all found or until 12 spins were reached for 2.5–3.5-year olds, 16 spins were reached for 3.5–4.5-year olds, or 20 spins were reached for 4.5–5.5-year olds. WM scores were calculated as a proportion of the number of stickers the child accurately selected compared to the total number of spins. Scores ranged from 0 to 1 with higher performance indicative of higher WM and low scores reflecting lower WM. Test–retest reliability on this task as administered to 2-year olds was significant (*r* = 0.59, *p* = 0.002; Lalonde and Holt, 2014).

##### Vocabulary

The Peabody Picture Vocabulary Test was used to assess receptive vocabulary (fourth ed.; PPVT-IV; Dunn and Dunn, 2007). The procedure for this test included an experimenter saying a word from the standardized list, and the child pointing to the picture that they thought corresponded with the word. The child would need to choose from four pictures when responding. If a child reached eight items from a block of 12 that were incorrect responses, then testing would stop. Otherwise, testing proceeded through the entire test. The results for each child were then compared to standardized scores.

##### Child Behavior Problems

The externalizing dimensional behavior problems subscale of the Child Behavior Checklist (CBCL; Achenbach and Rescorla, 2000) was completed by parents to assess behavioral maladjustment. The CBCL is a widely used 100-item questionnaire measure of socio-emotional and behavioral maladjustment appropriate for preschool age children. Parents rate their child’s behaviors over the past 2 months on a scale from 0 (“not true”) to 2 (“very true or often true”). Subscale total scores are calculated by summing raw parental responses for each subscale item, and z-scores are taken of these total summed scores. Higher scores reflect higher levels of behavioral maladjustment. Cronbach’s alpha for the externalizing dimensional behavior problems subscale was 0.80, consistent with published alpha level means of 0.76 for narrow subscales and 0.92 for broad constructs on the CBCL (Achenbach and Edelbrock, 1983).

##### Maternal Depressive Symptoms

We used the Center for Epidemiologic Studies Depression Scale (CES-D; Radloff, 1977) to measure primary caregiver depressive symptoms. This self-report questionnaire contains 20 items that assess depressive symptoms reported over the last week. The CES-D includes cognitive, emotional, behavioral, and positive affect items rated on a 4-point Likert scale from 0 (“rarely or none of the time/less than 1 day”) to 3 (“most or all of the time/5–7 days”). Global scores were formed by summing raw item scores, with higher scores representing higher depressive symptoms (range = 0–60). The Cronbach’s alpha for CES-D was 0.84, with published alphas ranging from 0.84 to 0.90 (Radloff, 1977).

##### Maternal Trait Anxiety

The A-Trait scale of the State–Trait Anxiety Inventory (STAI; Spielberger et al., 1970) was employed to assess trait anxiety in primary caregivers. This scale is comprised of 20 statements depicting general feelings scored by participants on a 4-point Likert scale from 1 (“not at all”) to 4 (“very much so”). Higher scores represent higher levels of trait anxiety. The Cronbach’s alpha of 0.90 for the STAI A-Trait scale in our sample was in accordance with the published range of 0.86 to 0.95 (Spielberger, 1989).

##### Data Reduction of the Lab-TAB IC Episodes

Data reduction was performed in order to create a composite Lab-TAB IC episode score data were reduced using a principle component analysis with oblique rotations on the behavioral variables from the two IC episodes of Lab-TAB. “snack delay” included two IC variables that presented as “fidgeting behavior” and “self-distracting behavior.” Both variables were concluded as important parts of child IC, and coded behaviors with high factor loadings (eigenvalue over 0.60) on either “fidgeting behavior” or “self-distracting behavior” were standardized and a mean composite snack delay IC score was calculated with higher values representing higher IC. The gift variables behaviors included fidgeting/self-distracting behavior and “impulsivity to open the gift.” Again, coded behavior variables that had high factor loadings on fidgeting/self-distracting behavior or “impulsivity to open the gift” were standardized and averaged to produce a composite gift IC count.

### Data Analysis

Preliminary analyses included calculating descriptive statistics and analyzing gender differences using *t*-tests and Cohen’s *d*. We used first order correlations to examine relations between child IC, WM, receptive vocabulary, behavior problems, and maternal depression and anxiety. To further examine the interrelationships among these variables and correct the underestimated standard errors due to the nested data structure (siblings within families), we employed the design-based multilevel structural equation model by using the Type = Complex routine in M*plus 8.5* (Muthén and Muthén, 1998-2020; Wu and Kwok, 2012). This routine adopted the maximum likelihood model estimation with robust standard errors and generated unbiased parameter estimates.

## Results

### Descriptive Statistics and Correlations

Table 1 includes all descriptive statistics (means and standard deviations) and gender differences. There were significant gender differences for several measures of IC, and there were none for WM, externalizing behavior problems, receptive vocabulary, and maternal depression and anxiety. Males had higher observer-rated hyperactivity and impulsivity, and females had higher observer-rated attention and Stroop-IC. Significant gender difference effect sizes ranged from 0.37 to 0.56 of a standard deviation. Correlations among the study variables are presented in Table 2. Excepting IC-Stroop and Lab-TAB snack delay, all of our IC measures were correlated with one another in the expected directions. All of the IC measures except Lab-TAB snack delay were positively correlated with WM, and with receptive vocabulary (except Lab-TAB snack delay and observer-rated hyperactivity), and WM and receptive vocabulary were also positively correlated. All IC variables, WM, and receptive vocabulary were significantly related to externalizing behavior problems in the expected direction. Generally, children with higher IC, WM, and receptive vocabulary had lower levels of behavioral maladjustment. Lower parent-rated IC and higher levels of externalizing behavior problems were positively associated with maternal depression and anxiety (lower receptive vocabulary level was related to depression only).

**Table 1:** Descriptive statistics and gender differences among study variables.

		Girls (*n* = 96)	Boys (*n* = 102)				
Variable	Overall Mean (*SD*)	Mean *(SD)*	Mean *(SD)*	*t* value	df	*p* value	Cohen’s *d*
1. IC: Observer attention	3.45 (0.95)	3.70 (0.82)	3.23 (1.01)	−3.59	196	0.000^**^	0.51
2. IC: Observer hyperactivity	2.59 (1.15)	2.29 (0.91)	2.86 (1.29)	3.59	196	0.000^**^	0.51
3. IC: Observer impulsivity	2.82 (0.98)	2.55 (0.77)	3.08 (1.09)	3.93	195	0.000^**^	0.56
4. IC: Lab-TAB snack delay	0.00 (0.67)	0.08 (0.63)	−0.07 (0.70)	−1.53	194	0.128	0.23
5. IC: Lab-TAB gift	−0.02 (0.62)	−0.04 (0.62)	0.00 (0.62)	0.565	196	0.573	0.06
6. IC: Stroop	7.82 (3.44)	8.44 (3.41)	7.20 (3.37)	−2.41	172	0.017^*^	0.37
7. IC: TBAQ parent report	0.00 (1.00)	0.11 (1.0)	−0.10 (1.0)	−1.45	196	0.149	0.21
8. WM: Spin-the-Pots	7.49 (1.96)	7.56 (2.04)	7.43 (1.89)	−0.44	190	0.660	0.07
9. Receptive vocabulary: PPVT	107.66 (14.26)	107.33 (13.58)	107.99 (14.95)	0.322	191	0.747	0.05
10. CBCL: Externalizing	12.85 (7.63)	12.43 (7.36)	13.26 (7.89)	0.749	191	0.455	0.11
11. CES: Maternal depression	0.00 (1.00)	0.06 (1.09)	−0.06 (0.90)	−0.798	196	0.426	0.12
12. STAI: Maternal anxiety	0.00 (1.00)	0.12 (1.02)	−0.11 (0.98)	−1.60	196	0.112	0.23

* *p* < 0.05;

** *p* < 0.01.

**Table 2:** Correlations among study variables.

Variable	1	2	3	4	5	6	7	8	9	10	11	12
1. IC: Observer attention	—											
2. IC: Observer hyperactivity	−0.50^**^	—										
3. IC: Observer impulsivity	−0.63^**^	0.68^**^	—									
4. IC: Lab-TAB snack delay	0.28^**^	−0.28^**^	−0.29^**^	—								
5. IC: Lab-TAB gift	0.30^**^	−0.25^**^	−0.33^**^	0.32^**^	—							
6. IC: Stroop	0.44^**^	−0.27^**^	−0.33^**^	0.13	0.18^*^	—						
7. IC: TBAQ parent report	0.36^**^	−0.18^*^	−0.32^**^	0.20^**^	0.27^**^	0.26^**^	—					
8. WM: Spin-the-Pots	0.39^**^	−0.15^*^	−0.28^**^	0.07	0.21^**^	0.35^**^	0.34^**^	—				
9. Receptive vocabulary: PPVT	0.31^**^	−0.14	−0.16^*^	0.09	0.16^*^	0.19^*^	0.37^**^	0.24^**^	—			
10. CBCL: Externalizing	−0.29^**^	0.25^**^	0.32^**^	−0.13	−0.13	−0.17^*^	−0.54^**^	−0.19^**^	−0.18^*^	—		
11. CES: Maternal depression	−0.06	0.06	0.10	0.02	−0.02	0.05	−0.29^**^	0.00	−0.22^**^	0.24^**^	—	
12. STAI: Maternal anxiety	0.08	−0.01	−0.06	0.05	−0.01	0.05	−0.28^**^	0.07	−0.10	0.23^**^	0.54^**^	—

* *p* < 0.05;

** *p* < 0.01.

### Results for Hypothesized Model

In the hypothesized model, we further examined the interrelationships among IC temperament variables, Stroop task, WM, and receptive vocabulary, controlling for age, gender, externalizing behaviors, maternal depression and anxiety, and the parent-rater variance (the multi-method effect). Even though the overall model chi-square test was significant [chi (56) = 80.652, *p* < 0.05], all the fit indices (SRMR = 0.049, RMSEA = 0.047, and CFI = 0.961) still indicated that our hypothesized model fits adequately to the data.

**Figure 1:**
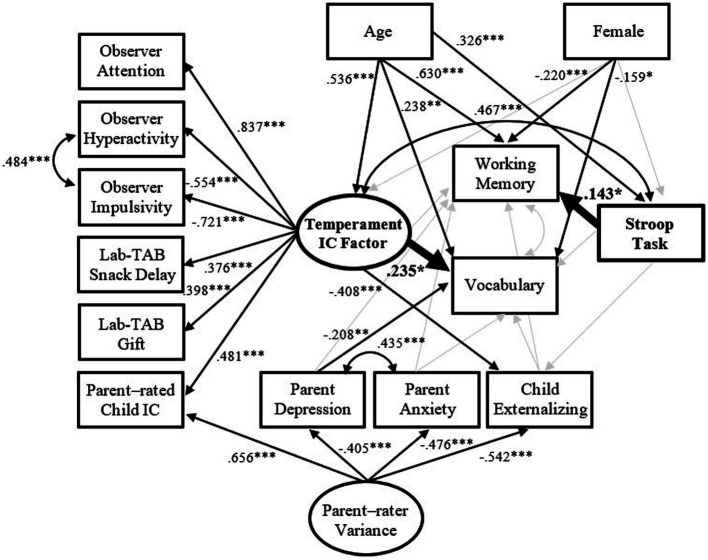
The results of hypothesized model. Gray paths are not statistically significant. Values are standardized path coefficients. ^*^p < 0.05; ^**^p < 0.01; and ^***^p < 0.001.

As shown in Figure 1, over and above the control variables, the IC Temperament factor, composed of a set of six temperament IC measures, showed a positive effect on receptive vocabulary, while Stroop task positively predicted WM. Meanwhile, the IC Temperament factor and Stroop task were positively correlated with each other. As expected, the IC Temperament factor, Stroop task, WM, and receptive vocabulary were positively related to age. The higher the IC Temperament factor was associated with fewer externalizing behavior problems. Maternal depression presented a negative effect on receptive vocabulary. Results of this model also revealed that females showed lower levels of WM and receptive vocabulary than males. Overall, the IC Temperament factor and other covariates together accounted for 22.5% of the variance in receptive vocabulary, whereas Stroop task and other controlled variables could explain 49.8% of the variance in WM.

## Discussion

The principal aim of this investigation was to study relations between preschool IC, WM, receptive vocabulary, behavior problems, and maternal depression and anxiety in a multi-theoretical and multi-method family study design. Much previous research on IC is theoretically based on either the EF or temperament perspective, employing methods and assessment techniques that reflect one or the other. This investigation measured IC using both temperament and EF approaches and included parent ratings, observer ratings, and laboratory tasks. In addition, a laboratory-based assessment of preschool WM and a standardized nonverbal vocabulary test was used, and we considered child age and gender, as well as maternal depression and anxiety as important covariates. Our mean-level gender analyses showed significant differences in several IC measures, but not in the other child or parent variables. Males had higher impulsivity and hyperactivity scores, while females had higher attention and IC-Stroop scores. Correlational analyses indicated that most all of the IC measures were significantly associated with one another, WM and receptive vocabulary. IC, WM, and receptive vocabulary were also correlated with externalizing behavior problems, such that preschoolers with higher IC, WM, and receptive vocabulary had fewer behavioral issues. Lower parent-rated IC and higher levels of externalizing were related to maternal depression and anxiety (lower receptive vocabulary level was linked to depression). Our SEM analyses provide some specificity by indicating that the IC Temperament factor had a positive effect on receptive vocabulary, and IC-Stroop was positively associated with WM, accounting for 22.5% of the variance in receptive vocabulary and 49.8% of WM variance, respectively. Both IC factors, WM, and receptive vocabulary were positively linked to age. The IC Temperament factor predicted fewer externalizing behavior problems, maternal depression had a negative effect on receptive vocabulary, and females showed lower WM and receptive vocabulary than males.

As expected based on EF theory and previous literature, preschool IC was significantly associated with WM and receptive vocabulary (e.g., Bronson, 2000; Blair, 2002; Engle and Kane, 2004; McClelland et al., 2007; Wolfe and Bell, 2007; Unsworth et al., 2012). In addition, preschool IC, WM and receptive vocabulary were significant correlates of externalizing behavior problems consistent with previous studies in childhood (e.g., Eisenberg et al., 2001, 2004; Goos et al., 2009; Gagne et al., 2011). In most cases, the correlational IC findings were similar across both parent and observed preschool IC, and across temperament and EF ratings of IC (e.g., Nigg et al., 1999; Olson et al., 1999; Eisenberg et al., 2001, 2005; Murray and Kochanska, 2002; Riggs et al., 2003; Hughes and Ensor, 2008; Gagne et al., 2011, 2018). The IC measure employing a snack delay task did not relate as strongly to the variables under study, suggesting that food-based impulse control tasks may not be as salient as other IC tasks in relation to WM, receptive vocabulary, and behavior problems. The current study reinforces previous EF research by showing clear links between WM, receptive vocabulary, and our multiple measures of IC. To our knowledge, this is the first investigation to include a parent-rating of IC, two Lab-TAB IC episodes, a Stroop task, and three post-visit experimenter ratings of IC as well as measures of WM and receptive vocabulary. Finally, SEM analyses take the age and gender of the child, as well as primary caregiver depression and anxiety into account, allowing for greater specificity in identifying relations between study variables. After accounting for these influences, there is a clear delineation between temperament and EF assessments of IC and links to WM, receptive vocabulary, and behavioral maladjustment.

The gender differences observed were fairly consistent with temperament theory, except for those found in the SEM analyses indicating that females had lower WM and receptive vocabulary. Although the effects were significant, they were not strong and could be a result of the multiple variables accounted for in the model. The correlational and SEM findings indicate that our wide age range spanning the preschool period is an important consideration when studying the early development of childhood IC, WM, and receptive vocabulary. The 2.5–5.5-year age range permitted the modeling of age effects from toddlerhood through late preschool. Overall, older children had higher IC, WM, and receptive vocabulary and lower levels of behavior problems, consistent with the developmental literature. Our SEM analyses bolstered the correlational results, showing that child age significantly predicted IC Temperament and Stroop factors, WM, and receptive vocabulary. These findings suggest a positive trajectory in the development of temperament-based IC, EF, and receptive vocabulary across the toddler-late preschool period.

In two previous papers, maternal depression was significantly related to parent-rated negative temperament traits and externalizing and ADHD behavior problems in this sample (Gagne et al., 2013b, 2018). In addition, maternal anxiety symptoms were positively and negatively associated with the CBCL externalizing and ADHD subscales, and parent ratings of IC, respectively (Gagne et al., 2018). These findings suggest that mothers with more depression and anxiety symptoms hold more negative views of their children’s IC and behavioral maladjustment. Interestingly, there was no significant covariance between maternal depression and anxiety and the observed IC variables. This pattern indicates that a rater effect is operating that reflects that primary caregiver depression and anxiety negatively influence caregiver perceptions of preschooler IC and behavioral maladjustment, artificially amplifying correlations between caregiver-reported mental health and child behavior. The current study found a similar pattern of results with maternal mental health symptoms significantly correlated to mother-rated IC and externalizing, but not with any observed ratings of IC or WM (there was a single significant correlation between maternal depression and receptive vocabulary replicated in the SEM model). This overall pattern highlights the discrepancy between parent and observed temperament and EF measures, and the issue of parent-rater variance contributing to inflated effects when all variables in an analysis are parent-reported.

As previously mentioned, our SEM analyses provide some specificity for how temperament and EF conceptions of IC (our factors in the model) relate to WM, receptive vocabulary, externalizing, and maternal mental health. Our correlational results were expected based on previous research, but the SEM results provide novelty and impact by disentangling the two theoretical approaches. The unique effect of IC-Stroop on WM was not surprising because both are considered EFs and previous research has found similar links between the two constructs. The IC Temperament factor had a unique effect on receptive vocabulary and externalizing, which was somewhat surprising. Since we often assume receptive vocabulary has a strong cognitive component and some studies have indicated as much, it would be expected that IC-Stroop would be associated with receptive vocabulary as well. Studies of IC from the EF perspective have also shown consistent relations with externalizing. We wondered why the IC Temperament factor would be significantly related to receptive vocabulary and externalizing while the Stroop was not? One interesting view would be that there may some combination of cognitive and socio-emotional IC influences that are optimal for learning receptive vocabulary and exhibiting lower externalizing during this phase of development. A related potential explanation is based on the analysis, as the paths that are loading highest on the IC Temperament factor are observer-rated attention and impulsivity. Therefore, attention (cognitive) and impulsivity (perhaps some socio-emotional component) are important precursors for optimal receptive vocabulary acquisition and self-regulation. The development of attention skills and self-regulation (controlling impulsivity) could represent developmental “cascades” (e.g., Wade et al., 2016) whereby these early emerging primary traits are essential for engagement, learning, and behavioral adjustment later in development.

The primary limitations of this investigation include the TEXAS Family Study sample size composition, and wide age range, lack of paternal mental health data, three different age-related versions of IC-Stroop tasks, single measures of WM and receptive vocabulary, a single parent-rated measure of externalizing, and only one IC measure representative of the EF perspective. Though 100 pairs of siblings and primary caregivers is an adequate sample size to perform our analyses, a larger sample might increase statistical power. Siblings allow us to model some partner effects but they are not independent data points. Therefore, we are constrained to running data analyses that account for dyadic effects. Relatedly, our sample was mostly white families with middle SES or higher, reducing generalizability. Future IC studies should utilize more diverse and representative samples. In addition, many investigations of preschool development measure behavior at a specific age or use a more restricted age range. However, our slightly wide age range provided us the option to study age effects across the preschool period with multiple IC assessments, and we accounted for age in our SEM models. In two of our current studies, we are measuring IC at one age (age 3) or with a more restricted age range (3–5) allowing for better age-appropriate assessment and less participant variance. Access to paternal mental health data could also enhance studies of the early development of IC and behavior problems. Unfortunately, there were limited resources available in this study and we were only able to assess primary caregivers. Lastly, this is one of very few studies that assessed IC using both the EF and temperament approaches. The use of three separate Stroop tasks can also be viewed as a limitation. Although we modeled age effects in our SEM analyses, future studies of early IC might benefit from the use of a single measurement. Although we had several temperament measures of IC, there was only one basic Stroop task representing EF and only single measures of WM, receptive vocabulary, and externalizing. Although the use of unitary measures to investigate a construct is a limitation, all three of these measures are standard, laboratory-based measures (as opposed to parent-rated questionnaires). In our current investigations, we are using a computerized Go/NoGo task with three-year-old children and will potentially use Flanker tasks in another with older children as well as Stroops. In addition, we will employ a multi-method approach whenever possible in examining constructs, such as WM and receptive vocabulary.

This study addresses limitations of the current literature by examining relations between both temperament and EF conceptions of IC, WM, receptive vocabulary, and externalizing in preschoolers. The inclusion on multiple measures of IC, maternal depression and anxiety, and consideration of gender and age effects further contextualizes our findings. Although both EF and temperament measures of IC evinced somewhat comparable patterns of correlational findings with WM, receptive vocabulary, and child behavioral maladjustment, the SEM analyses provide some interesting specificity. The IC temperament effects predicted receptive vocabulary and externalizing, and the Stroop predicted WM. Parent-rated IC was linked to caregiver depression and anxiety, but observed IC variables, WM, and receptive vocabulary were not. These results suggest that caregiver depression and anxiety symptoms relate to caregiver observations of child IC and receptive vocabulary, but not to IC and WM measured in the laboratory. This rater effect has critical consequences for future studies of early emerging IC and other dimensions of child temperament, EF, and behavioral problems, including the possibility that caregiver expectancies of preschool IC will influence child behavior later in development, as well as the specter of parent-rater biases. Ultimately, including observational measures in IC assessment strategies reflecting both EF and temperament is worthwhile as laboratory observations are not vulnerable to parent-rater biases and may confer higher specificity. In addition, a multi-theoretical and multi-method assessment approach may contribute to more robust and precise conclusions. Our future studies focus on a longitudinal follow-up with this sample in early elementary school to assess the developmental trajectories of preschool IC, WM, and receptive vocabulary in the family context, and ongoing studies comparing different types of observed assessments of IC at different ages in preschool along with concurrently developing neurophysiology as well as school readiness and transition.

## Data Availability Statement

The raw data supporting the conclusions of this article will be made available by the authors, without undue reservation.

## Ethics Statement

The studies involving human participants were reviewed and approved by the Texas A&M University IRB. Written informed consent to participate in this study was provided by the participants’ legal guardian/next of kin.

## Author Contributions

JG and KB: study conception, data analysis plan, data analyses, and writing. C-NC: data analysis plan, data analyses, and writing. ON: writing. O-MK: data analysis plan and writing. All authors contributed to the article and approved the submitted version.

## Conflict of Interest

The authors declare that the research was conducted in the absence of any commercial or financial relationships that could be construed as a potential conflict of interest.

## Publisher’s Note

All claims expressed in this article are solely those of the authors and do not necessarily represent those of their affiliated organizations, or those of the publisher, the editors and the reviewers. Any product that may be evaluated in this article, or claim that may be made by its manufacturer, is not guaranteed or endorsed by the publisher.

## Footnotes

^1^Goldsmith, H. H., Reilly, J., Lemery, K. S., Longley, S., Prescott, A. (1995). Preschool Laboratory Temperament Assessment Battery. Unpublished instrument, University of Wisconsin.

## References

[ref1] Achenbach T. M. Edelbrock C. (1983). Manual for the Child Behavior Checklist and Revised Behavior Profile. Burlington: University of Vermont Department of Psychiatry.

[ref2] Achenbach T. M. Rescorla L. A. (2000). Manual for the ASEBA Preschool Forms & Profiles: An Integrated System of Multi-Informant Assessment; Child Behavior Checklist for Ages 1 1/2-5; Language Development Survey; Caregiver-Teacher Report Form. Burlington: University of Vermont.

[ref3] Baddeley A. D. Hitch G. J. (1994). Developments in the concept of working memory. Neuropsychology 8, 485–493. 10.1037/0894-4105.8.4.485

[ref4] Biederman J. Hirshfeld-Becker D. R. Rosenbaum J. F. Hérot C. Friedman D. Snidman N.. (2001). Further evidence of association between behavioral inhibition and social anxiety in children. Am. J. Psychiatr. 158, 1673–1679. 10.1176/appi.ajp.158.10.1673 11579001

[ref5] Blair C. (2002). School readiness: integrating cognition and emotion in a neurobiological conceptualization of children’s functioning at school entry. Am. Psychol. 57, 111–127. 10.1037/0003-066X.57.2.111 11899554

[ref6] Bronson M. B. (2000). Self-Regulation in Early Childhood: Nature and Nurture. New York: Guilford Press.

[ref7] Cain K. Oakhill J. Bryant P. (2004). Children’s reading comprehension ability: concurrent prediction by working memory, verbal ability, and component skills. J. Educ. Psychol. 96, 31–42. 10.1037/0022-0663.96.1.31

[ref8] Caspi A. Roberts B. W. Shiner R. L. (2005). Personality development: stability and change. Annu. Rev. Psychol. 56, 453–484. 10.1146/annurev.psych.55.090902.141913, PMID: 15709943

[ref9] Chasiotis A. Kiessling F. Hofer J. Campos D. (2006). Theory of mind and inhibitory control in three cultures: conflict inhibition predicts false belief understanding in Germany, Costa Rica and Cameroon. Int. J. Behav. Dev. 30, 249–260. 10.1177/0165025406066759

[ref10] Cromley J. G. Azevedo R. (2007). Testing and refining the direct and inferential mediation model of reading comprehension. J. Educ. Psychol. 99, 311–325. 10.1037/0022-0663.99.2.311

[ref12] Diamond A. (2013). Executive functions. Annu. Rev. Psychol. 64, 135–168. 10.1146/annurev-psych-113011-143750, PMID: 23020641 PMC4084861

[ref13] Dunn L. M. Dunn D. M. (2007). Peabody Picture Vocabulary Test. 4th *Edn*. Minneapolis, MN: Pearson Education.

[ref14] Edhborg M. Seimyr L. Lundh W. Widström A.-M. (2000). Fussy child—difficult parenthood? Comparisons between families with a “depressed” mother and non-depressed mother 2 months postpartum. J. Reprod. Infant Psychol. 18, 225–238. 10.1080/713683036

[ref15] Eisenberg N. Cumberland A. Spinrad T. L. Fabes R. A. Shepard S. A. Reiser M.. (2001). The relations of regulation and emotionality to children’s externalizing and internalizing problem behavior. Child Dev. 72, 1112–1134. 10.1111/1467-8624.00337, PMID: 11480937

[ref16] Eisenberg N. Spinrad T. L. Fabes R. A. Reiser M. Cumberland A. Shepard S. A.. (2004). The relations of effortful control and impulsivity to children’s resiliency and adjustment. Child Dev. 75, 25–46. 10.1111/j.1467-8624.2004.00652.x, PMID: 15015673 PMC1785300

[ref17] Eisenberg N. Zhou Q. Spinrad T. L. Valiente C. Fabes R. A. Liew J. (2005). Relations among positive parenting, children’s effortful control, and externalizing problems: A three-wave longitudinal study. Child Dev. 76, 1055–1071. 10.1111/j.1467-8624.2005.00897.x, PMID: 16150002 PMC1351058

[ref18] Engle R. W. Kane M. J. (2004). “Executive attention, working memory capacity, and a two-factor theory of cognitive control,” in The Psychology of Learning and Motivation: Advances in Research and Theory. *Vol*. 44. ed. Ross B. H. (New York, NY: Elsevier Science), 145–199.

[ref19] Florit E. Roch M. Altoe G. Levorato M. C. (2009). Listening comprehension in preschoolers: The role of memory. Br. J. Dev. Psychol. 27, 935–951. 10.1348/026151008X397189 19994487

[ref20] Gagne J. R. (2017). Self-control in childhood: A synthesis of perspectives and focus on early development. Child Dev. Perspect. 11, 127–132. 10.1111/cdep.12223

[ref21] Gagne J. R. Chang C. Fang H. A. Spann C. A. Kwok O. (2018). A multi-method study of inhibitory control and behavior problems in preschoolers. Infant Child Dev. 28:e2115. 10.1002/icd.2115

[ref22] Gagne J. R. Goldsmith H. H. (2011). A longitudinal analysis of anger and inhibitory control in twins from 12–36 months of age. Dev. Sci. 14, 112–124. 10.1111/j.1467-7687.2010.00969.x, PMID: 21159093 PMC3049157

[ref23] Gagne J. R. Miller M. M. Goldsmith H. H. (2013a). Early—but modest—gender differences in focal aspects of childhood temperament. Personal. Individ. Differ. 55, 95–100. 10.1016/j.paid.2013.02.006, PMID: 24958978 PMC4064677

[ref24] Gagne J. R. Saudino K. J. (2010). Wait for it! A twin study of inhibitory control in early childhood. Behav. Genet. 40, 327–337. 10.1007/s10519-009-9316-6, PMID: 19936910 PMC2854273

[ref25] Gagne J. R. Saudino K. J. (2016). The development of inhibitory control in early childhood: A twin study from 2-3 years. Dev. Psychol. 52, 391–399. 10.1037/dev0000090, PMID: 26784384 PMC4839189

[ref26] Gagne J. R. Saudino K. J. Asherson P. (2011). The genetic etiology of inhibitory control and behavior problems at 24 months of age. J. Child Psychol. Psychiatry 52, 1155–1163. 10.1111/j.1469-7610.2011.02420.x, PMID: 21627653 PMC3184216

[ref27] Gagne J. R. Spann C. A. Prater J. C. (2013b). Parent depression symptoms and child temperament outcomes: A family study approach. J. Appl. Biobehav. Res. 18, 175–197. 10.1111/jabr.12013

[ref28] Garon N. Bryson S. E. Smith I. M. (2008). Executive function in preschoolers: A review using an integrative framework. Psychol. Bull. 134, 31–60. 10.1037/0033-2909.134.1.31, PMID: 18193994

[ref29] Goldsmith H. H. (1996). Studying temperament via construction of the toddler behavior assessment questionnaire. Child Dev. 67, 218–235. 10.2307/1131697, PMID: 8605830

[ref30] Goldsmith H. H. Lemery K. S. Essex M. J. (2004). “Temperament as a liability factor for childhood behavioral disorders: the concept of liability,” in Behavior Genetics Principles: Perspectives in Development, Personality, and Psychopathology. Decade of Behavior (Washington, DC, US: American Psychological Association), 19–39.

[ref31] Goos L. M. Crosbie J. Payne S. Schachar R. (2009). Validation and extension of the endophenotype model in ADHD patterns of inheritance in a family study of inhibitory control. Am. J. Psychiatr. 166, 711–717. 10.1176/appi.ajp.2009.08040621 19448185

[ref32] Hill A. C. Laird A. R. Robinson J. L. (2014). Gender differences in working memory networks: A BrainMap meta-analysis. Biol. Psychol. 102, 18–29. 10.1016/j.biopsycho.2014.06.008, PMID: 25042764 PMC4157091

[ref33] Hughes C. (1998). Executive function in preschoolers: links with theory of mind and verbal ability. Br. J. Dev. Psychol. 16, 233–253. 10.1111/j.2044-835X.1998.tb00921.x

[ref34] Hughes C. Ensor R. (2005). Executive function and theory of mind in 2 year olds: a family affair? Dev. Neuropsychol. 28, 645–668. 10.1207/s15326942dn2802_5, PMID: 16144431

[ref35] Hughes C. Ensor R. (2007). Executive function and theory of mind: predictive relations from ages 2 to 4. Dev. Psychol. 43, 1447–1459. 10.1037/0012-1649.43.6.1447, PMID: 18020823

[ref36] Hughes C. Ensor R. (2008). Does executive function matter for preschoolers’ problem behaviors? J. Abnorm. Child Psychol. 36, 1–14. 10.1007/s10802-007-9107-6, PMID: 17914667

[ref37] Johnson J. Im-Bolter N. Pascual-Leone J. (2003). Development of mental attention in gifted and mainstream children: The role of mental capacity, inhibition, and speed of processing. Child Dev. 74, 1594–1614. 10.1046/j.1467-8624.2003.00626.x, PMID: 14669884

[ref38] Kälin S. Roebers C. M. (2021). Self-regulation in preschool children: factor structure of different measures of effortful control and executive functions. J. Cogn. Dev. 22, 48–67. 10.1080/15248372.2020.1862120

[ref39] Kochanska G. Murray K. Jacques T. Y. Koenig A. L. Vandegeest K. A. (1996). Inhibitory control in young children and its role in emerging internalization. Child Dev. 67, 490–507. 10.2307/1131828, PMID: 8625724

[ref40] Krull J. L. (2007). Using multilevel analyses with sibling data to increase analytic power: An illustration and simulation study. Dev. Psychol. 43, 602–619. 10.1037/0012-1649.43.3.602, PMID: 17484574

[ref41] Lalonde K. Holt R. F. (2014). Cognitive and linguistic sources of variance in 2-year-olds’speech-sound discrimination: a preliminary investigation. J. Speech Lang. Hear. Res. 57, 308–326. 10.1044/1092-4388(2013/12-0227), PMID: 24023371 PMC5600153

[ref11] Language and Reading Research Consortium, Currie N. K. Muijselaar M. M. L. (2019). Inference making in young children: The concurrent and longitudinal contributions of verbal working memory and vocabulary. J. Educ. Psychol. 111, 1416–1431. 10.1037/edu0000342

[ref42] Lerner M. D. Lonigan C. J. (2014). Executive function among preschool children: unitary versus distinct abilities. J. Psychopathol. Behav. Assess. 36, 626–639. 10.1007/s10862-014-9424-3, PMID: 25642020 PMC4306461

[ref43] Lin B. Liew J. Perez M. (2019). Measurement of self-regulation in early childhood: relations between laboratory and performance-based measures of effortful control and executive functioning. Early Child. Res. Q. 47, 1–8. 10.1016/j.ecresq.2018.10.004, PMID: 31223199 PMC6585984

[ref44] Lynch J. S. Van den Broek P. Kremer K. E. Kendeou P. White M. J. Lorch E. P. (2008). The development of narrative comprehension and its relation to other early reading skills. Read. Psychol. 29, 327–365. 10.1080/02702710802165416

[ref45] McClelland M. M. Cameron C. E. Connor C. M. Farris C. L. Jewkes A. M. Morrison F. J. (2007). Links between behavioral regulation and preschoolers’ literacy, vocabulary, and math skills. Dev. Psychol. 43, 947–959. 10.1037/0012-1649.43.4.947, PMID: 17605527

[ref46] Metcalfe J. Mischel W. (1999). A hot/cool system analysis of delay of gratification: dynamics of willpower. Psychol. Rev. 106, 3–19. 10.1037/0033-295X.106.1.3, PMID: 10197361

[ref47] Morra S. Panesi S. Traverso L. Usai M. C. (2018). Which tasks measure what? Reflections on executive function development and a commentary on Podjarny, Kamawar, and Andrews (2017). J. Exp. Child Psychol. 167, 246–258. 10.1016/j.jecp.2017.11.004, PMID: 29197781

[ref48] Murray K. T. Kochanska G. (2002). Effortful control: factor structure and relation to externalizing and internalizing behaviors. J. Abnorm. Child Psychol. 30, 503–514. 10.1023/A:1019821031523, PMID: 12403153

[ref49] Muthén L. K. Muthén B. (1998-2020). Mplus user’s Guide: Statistical Analysis With Latent Variables, User’s Guide. Los Angeles, CA: Muthén & Muthén.

[ref50] Nigg J. T. Quamma J. P. Greenberg M. T. Kusche C. A. (1999). A two-year longitudinal study of neuropsychological and cognitive performance in relation to behavioral problems and competencies in elementary school children. J. Abnorm. Child Psychol. 27, 51–63. 10.1023/A:1022614407893, PMID: 10197406

[ref51] Olson S. L. Schilling E. M. Bates J. E. (1999). Measurement of impulsivity: construct coherence, longitudinal stability, and relationship with externalizing problems in middle childhood and adolescence. J. Abnorm. Child Psychol. 27, 151–165. 10.1023/A:1021915615677, PMID: 10400061

[ref52] Panesi S. Morra S. (2020). Executive functions and mental attentional capacity in preschoolers. J. Cogn. Dev. 21, 72–91. 10.1080/15248372.2019.1685525

[ref53] Pesonen A. K. Räikkönen K. Heinonen K. Järvenpää A. L. Strandberg T. E. (2006). Depressive vulnerability in parents and their 5-year-old child’s temperament: A family system perspective. J. Fam. Psychol. 20, 648–655. 10.1037/0893-3200.20.4.648, PMID: 17176200

[ref54] Podsakoff P. M. MacKenzie S. B. Lee J.-Y. Podsakoff N. P. (2003). Common method biases in behavioral research: A critical review of the literature and recommended remedies. J. Appl. Psychol. 88, 879–903. 10.1037/0021-9010.88.5.879 14516251

[ref55] Radloff L. S. (1977). The CES-D scale: A self-report depression scale for research in the general population. Appl. Psychol. Meas. 1, 385–401. 10.1177/014662167700100306

[ref56] Rhoades B. L. Greenberg M. T. Domitrovich C. E. (2009). The contribution of inhibitory control to preschoolers’ social–emotional competence. J. Appl. Dev. Psychol. 30, 310–320. 10.1016/j.appdev.2008.12.012

[ref57] Riggs N. R. Blair C. B. Greenberg M. T. (2003). Concurrent and 2-year longitudinal relations between executive function and the behavior of 1st and 2nd grade children. Child Neuropsychol. 9, 267–276. 10.1076/chin.9.4.267.23513, PMID: 14972705

[ref58] Rothbart M. K. (1989). “Temperament and development,” in Temperament in Childhood. eds. Kohnstamm G. A. Bates J. E. Rothbart M. K. (Oxford, England: John Wiley & Sons), 187–247.

[ref59] Rothbart M. K. Ahadi S. A. Hershey K. L. (1994). Temperament and social behavior in childhood. Merrill-Palmer Q. 40, 21–39.

[ref60] Rothbart M. K. Bates J. E. (2006). “Temperament,” in Handbook of Child Psychology: Social, Emotional, and Personality Development. 6th *Edn*. *Vol*. 3. eds. Damon W. Lerner R. Eisenberg N. (New York: Wiley), 99–166.

[ref61] Saudino K. J. (2003). Parent ratings of infant temperament: lessons from twin studies. Infant Behav. Dev. 26, 100–107. 10.1016/S0163-6383(02)00171-6

[ref62] Saudino K. J. Carter A. S. Purper-Ouakil D. Gorwood P. (2008). The etiology of behavioral problems and competencies in very young twins. J. Abnorm. Psychol. 117, 48–62. 10.1037/0021-843X.117.1.48, PMID: 18266485 PMC4103163

[ref63] Schmitt S. A. Purpura D. J. Elicker J. G. (2019). Predictive links among vocabulary, mathematical language, and executive functioning in preschoolers. J. Exp. Child Psychol. 180, 55–68. 10.1016/j.jecp.2018.12.005, PMID: 30639768

[ref64] Smith E. E. Jonides J. (1999). Storage and executive processes in the frontal lobes. Science 283, 1657–1661. 10.1126/science.283.5408.1657, PMID: 10073923

[ref65] Spann C. A. Gagne J. R. (2016). Aggression in young siblings: associations with executive functions and maternal characteristics. J. Abnorm. Child Psychol. 44, 523–533. 10.1007/s10802-015-0042-7, PMID: 26084593

[ref66] Spielberger C. D. (1989). State–Trait Anxiety Inventory: A Comprehensive Bibliography. Palo Alto, CA: Consulting Psychologists Press.

[ref67] Spielberger C. D. Gorsuch R. L. Lushene R. E. (1970). State-Trait Anxiety Inventory. Palo Alto, CA: Consulting Psychologists Press.

[ref68] Thorell L. B. Wåhlstedt C. (2006). Executive functioning deficits in relation to symptoms of ADHD and/or ODD in preschool children. Infant Child Dev. 15, 503–518. 10.1002/icd.475

[ref69] Tiego J. Bellgrove M. A. Whittle S. Pantelis C. Testa R. (2019). Common mechanisms of executive attention underlie executive function and effortful control in children. Dev. Sci. 23:e12918. 10.1111/desc.12918, PMID: 31680377

[ref70] Unsworth N. Engle R. (2007). The nature of individual differences in working memory capacity: active maintenance in primary memory and controlled search in secondary memory. Psychol. Rev. 114, 104–132. 10.1037/0033-295X.114.1.104, PMID: 17227183

[ref71] Unsworth N. McMillan B. D. Brewer G. A. Spillers G. J. (2012). Everyday attention failures: An individual differences investigation. J. Exp. Psychol. Learn. Mem. Cogn. 38, 1765–1772. 10.1037/a0028075, PMID: 22468805

[ref72] Unsworth N. Robison M. K. (2017). The importance of arousal for variation in working memory capacity and attention control: A latent variable pupillometry study. J. Exp. Psychol. Learn. Mem. Cogn. 43, 1962–1987. 10.1037/xlm0000421, PMID: 28504528

[ref73] Usai M. C. Viterbori P. Traverso L. De Franchis V. (2014). Latent structure of executive function in five-and six-year-old children: A longitudinal study. Eur. J. Dev. Psychol. 11, 447–462. 10.1080/17405629.2013.840578

[ref74] Ventura J. N. Stevenson M. B. (1986). Relations of mothers’ and fathers’ reports of infant temperament, parents’ psychological functioning, and family characteristics. Merrill-Palmer Q. 32, 275–289.

[ref75] von Stauffenberg C. Campbell S. B. (2007). Predicting the early developmental course of symptoms of attention deficit hyperactivity disorder. J. Appl. Dev. Psychol. 28, 536–552. 10.1016/j.appdev.2007.06.011, PMID: 21836767 PMC3153376

[ref76] Wade M. Browne D. T. Plamondon A. Daniel E. Jenkins J. M. (2016). Cumulative risk disparities in children’s neurocognitive functioning: a developmental cascade model. Dev. Sci. 19, 179–194. 10.1111/desc.12302, PMID: 25845409

[ref77] Wiebe S. A. Espy K. S. Charak D. (2008). Using confirmatory factor analysis to understand executive control in preschool children: I. latent structure. Dev. Psychol. 44, 575–587. 10.1037/0012-1649.44.2.575, PMID: 18331145

[ref78] Wiebe S. Scheffield T. Nelson J. M. Clark C. A. Chevalier N. Espy K. (2011). The structure of executive function in 3-year-olds. J. Exp. Child Psychol. 108, 436–452. 10.1016/j.jecp.2010.08.008, PMID: 20884004 PMC3033982

[ref79] Wolfe C. D. Bell M. A. (2007). The integration of cognition and emotion during infancy and early childhood: regulatory processes associated with the development of working memory. Brain Cogn. 65, 3–13. 10.1016/j.bandc.2006.01.009, PMID: 17630061

[ref80] Wu J. Y. Kwok O. M. (2012). Using SEM to analyze complex survey data: A comparison between design-based single-level and model-based multilevel approaches. Struct. Equ. Model. Multidiscip. J. 19, 16–35. 10.1080/10705511.2012.634703

[ref81] Zelazo P. D. (2015). Executive function: reflection, iterative reprocessing, complexity, and the developing brain. Dev. Rev. 38, 55–68. 10.1016/j.dr.2015.07.001

[ref82] Zelazo P. D. Müller U. Frye D. Marcovitch S. (2003). The development of executive function in early childhood: VI. The development of executive function: cognitive complexity and control--revised. Monogr. Soc. Res. Child Dev. 68, 93–119. 10.1111/j.0037-976X.2003.00266.x 14723273

[ref83] Zhou Q. Chen S. H. Main A. (2012). Commonalities and differences in the research on children’s effortful control and executive function: A call for an integrated model of self-regulation. Child Dev. Perspect. 6, 112–121. 10.1111/j.1750-8606.2011.00176.x

